# Three-dimensional misfit between Ti-Base abutments and implants evaluated by replica technique

**DOI:** 10.1590/1678-7757-2020-0343

**Published:** 2020-11-30

**Authors:** Karina Bergamo Cardoso, Edmara Tatiely Pedroso Bergamo, Vitor De Moraes Cruz, Ilana Santos Ramalho, Lucas Fracassi De Oliveira Lino, Estevam Augusto Bonfante

**Affiliations:** 1 Universidade de São Paulo Faculdade de Odontologia de Bauru Departamento de Prótese e Periodontia Bauru Brasil Universidade de São Paulo, Faculdade de Odontologia de Bauru, Departamento de Prótese e Periodontia, Bauru, Brasil.; 2 Universidade de São Paulo Faculdade de Odontologia de Bauru Departamento de Dentística, Endodontia e Materiais Odontológicos Bauru Brasil Universidade de São Paulo, Faculdade de Odontologia de Bauru, Departamento de Dentística, Endodontia e Materiais Odontológicos, Bauru, Brasil.

**Keywords:** Dental implants, Dental abutments, X-ray microtomography

## Abstract

An important factor affecting the biomechanical behavior of implant-supported reconstructions is the implant-abutment misfit.

**Objective:**

This study evaluated the misfit between Ti-Base abutments and implants by means of polyvinyl siloxane replica technique using microcomputed tomography (μCT).

**Methodology:**

Volumetric and linear (central and marginal) gaps of four Ti-base abutments (n=10/group): (i) Odontofix LTDA (OD), (ii) Singular Implants (SING), (iii) EFF Dental Components (EFF), and (iv) Control Group (S.I.N implants) compatible with an implant system (Strong SW, S.I.N Implants) were measured using μCT reconstructed polyvinyl siloxane replicas.

**Results:**

The results showed significantly lower volume gap for Control S.I.N (0.67±0.29 mm^3^) and SING (0.69±0.28 mm^3^) Ti-base abutments relative to OD (1.42±0.28 mm^3^) and EFF groups (1.04±0.28 mm^3^) (p<0.033), without significant difference between them (p=0.936). While gap values were homogenous in the central region, EFF presented a significantly higher marginal gap. Accordingly, the Control S.I.N and Singular Ti-base abutments showed improved volumetric and marginal fit relative to Odontofix and EFF.

**Conclusion:**

The method of manufacturing abutments influenced the misfit at the implant-abutment interface.

## Introduction

The increasing use of intraoral scanning and CAD-CAM technologies has challenged conventional fabrication procedures for prostheses for their expedited and patient-centered preference that supports a complete digital workflow, especially in dental implantology.^1^^,^^2^ Implant abutments that are tailored for CAD-CAM use, such as Ti-base, allow for digital design and milling of customized restorations to be extraorally cemented and screwed to the implant.^3^ The advantages of this technique include emergency profile customization, time efficiency with reduced costs, hybrid retention mechanism (cemented and screwed) that allows excess cement removal, and improved photocuring of the restoration margins prior to screwing.^4^^,^^5^

An important factor affecting the survival of implant-supported reconstructions is the fit at the implant-abutment interface, given that the clamping forces of such surfaces are maximized and most stable when the smallest gaps are present. Two-dimensional microscopic methods are discouraged to characterize the implant-abutment interface since only the margins are depicted, whereas methods measuring volume by microcomputed tomography (μCT), for instance, may provide a more thorough characterization of the overall interface misfit. Such methods are relevant since several implant abutments are compatible among brands, but may present different levels of volumetric misfit.^6^ The direct scanning of the implant-abutment interface by μCT is not only time consuming but also expensive. Therefore, this study measured the volumetric and linear gaps between Ti-base abutments of different brands to the implant interface using μCT by an alternate indirect method using a polyvinyl siloxane replica technique. The postulated null hypothesis was that compatible Ti-base abutments would present similar gap at the implant-abutment interface compared to proprietary implant-abutment assemblies.

## Methodology

Forty external hexagon implants (4.1x10 mm, Strong SW; S.I.N implants) were divided into 4 groups according to Ti-base abutment company (n=10/group): (i) OD (Odontofix LTDA, Ribeirão Preto, SP, Brazil), (ii) SING (Singular Implants, Parnamirim, RN, Brazil), (iii) EFF (EFF Dental Components, São Paulo, SP, Brazil), and (iv) Control Group (S.I.N implants, São Paulo, SP, Brazil).

The implants were embedded in acrylic resin to allow Ti-base abutment torqueing. Replicas of the gap between Ti-base and implant interface were produced by filling the implant platform with regular body polyvinyl siloxane impression material (Express XT Regular Body; 3M Oral Care) while the Ti-base abutments were torqued (32 N.cm) with a digital wrench (Tohnichi BTG150CN- S; Tohnichi America).^5^

**Figure 1:**
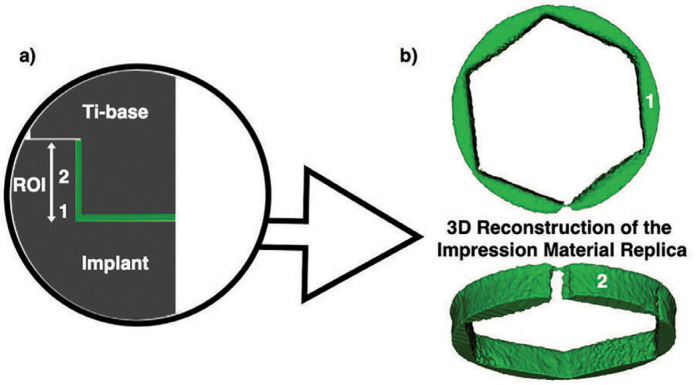
Schematic illustration of the silicone impression material inserted into the implant (a). To determine similar volume analysis across groups, the region of interest (ROI) was defined by cropping the replica image from the implant platform to the hexagon height. ROIs for linear marginal (1) and central (2) measurements are also depicted. 3D reconstruction of the gap replica (b)

The silicon replicas were individually scanned using a microcomputed tomography scanner (μCT X-Ray microfocus CT scanner; SkyScan). All data were exported in DICOM format and imported into Slicer (3D Slicer 4.10.2) for the 3D rendering process. After 3D reconstruction, the same software was used to measure the gap at the implant-abutment interface, which was represented by the thickness of the regular-body polyvinyl siloxane. To ensure that the replica specimens of different groups were all measured equally, the region of interest (ROI) was defined by cropping the replica image from the implant platform to the hexagon height. A uniform threshold for silicone was determined across all groups using the Otsu algorithm ROIs. After 3D reconstruction of the silicone replica, the volume of misfit as well as linear marginal and central gaps in a two-dimensional section were calculated using the “Segment Statistics” software function (Figure 1).^5^

Data normality was checked using Shapiro-Wilk's test (p>0.05). Volume data were statistically evaluated through one-way analysis of variance, whereas linear data through two-way analysis of variance. Post-hoc comparisons were performed by Tukey test (p<0.05). Data are presented as a function of mean and the corresponding 95% confidence interval.

## Results

**Figure 2:**
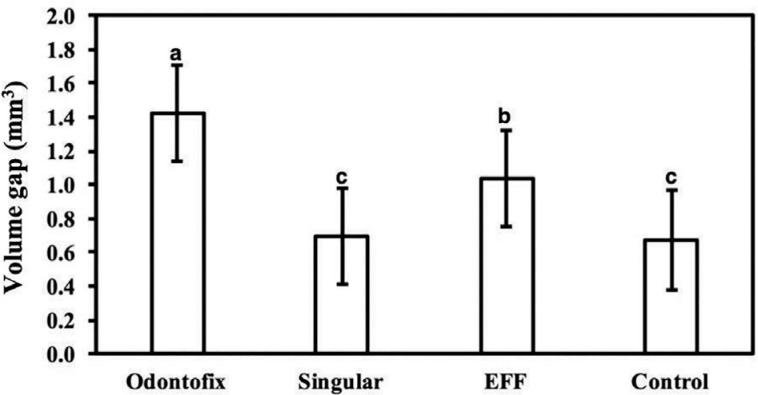
Volumetric misfit between Ti-base abutments and implant interface. The data are presented as a function of mean and 95% CI, and different letters indicate statistically significant difference

**Figure 3:**
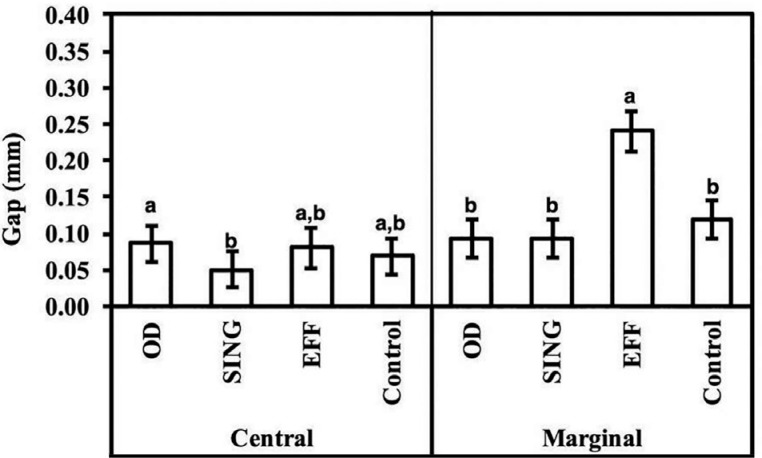
Qualitative volumetric misfit differences between Ti-base abutments and implants in the μCT reconstructed replicas of OD (a), SING (b), EFF (c), and Control (d)

The Control S.I.N (0.67±0.29 mm^3^) and SING (0.69±0.28 mm^3^) Ti-base abutments exhibited a significantly lower volume gap relative to OD (1.42±0.28 mm^3^) and EFF groups (1.04±0.28 mm^3^) (p<0.033), without significant difference between them (p=0.936) (Figures 2 and 3).

**Figure 4:**
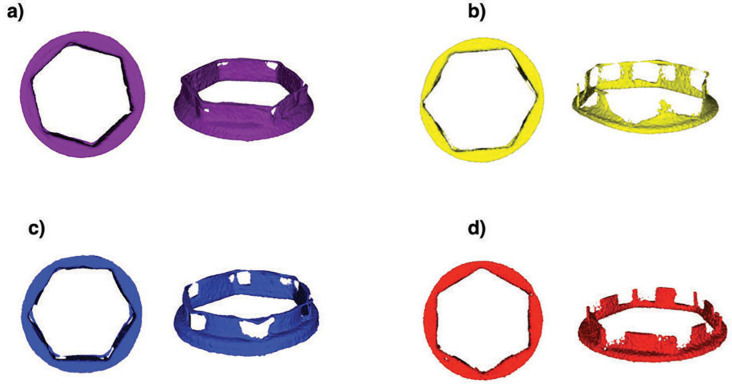
Linear two-dimensional gap measured for various Ti-base abutments at the central and marginal region. The data are presented as a function of mean and 95% CI, and different letters indicate statistically significant difference

Two-dimensional measurements of gap values between Ti-base and implant at the central (ROI 1) demonstrated significantly lower values for the SING group (0.050±0.025 mm) relative to OD (0.086±0.025 mm) (p<0.049); with no significant difference for all other pairwise comparisons (p>0.109). The marginal misfit (ROI 2) was significantly higher for the EFF (0.240±0.027 mm) group relative to others (p<0.001). In fact, a higher range in the mean gap values difference between Ti-base groups was observed for the marginal measurement (up to 0.14 mm) relative to the central misfit (~0.03 mm) (Figure 4).

## Discussion

The current study investigated the volumetric and linear gaps at the Ti-base abutment and implant interface using microcomputed tomography (μCT). Overall, the data demonstrated lower volumetric and two-dimensional marginal gaps for Control (S.I.N Implants) and Singular Ti-bases relative to Odontofix and EFF. Thus, the postulated null hypothesis that the Ti-base of different companies would present similar gap was rejected. This fact may lie on the industrial milling parameters and consequently the accuracy associated with the fabrication of Ti-base abutments, mainly for companies that have an implant-abutment interface compatible with several other implant companies. Compatibility generally implies a higher fit tolerance, meaning that non-proprietary abutments must present space to fit a variety of implant brands manufactured by different milling units and strategies.

The presence of gaps in the implant-abutment connection has previously shown to have a detrimental effect on biomechanical behavior through irregular stress distribution along the components.^5^^,^^6^ Higher misfit values between the components have been related to an increased stress concentration in the connection structures and in the surrounding tissues, where abutment screw fracture was the mostly reported technical complication with a cumulative failure rate of 10.4% over 5 years and a twofold increase after 10 years, 20.4%.^7^^,^^8^ Moreover, biological concerns have been raised regarding the presence of micro gaps in the implant-abutment connection that were related to bacterial colonization, which can induce peri-implant bone resorption and affect the long-term health of peri-implant tissues.^8^ Therefore, the clinical selection of an appropriate abutment may affect long-term implant-supported prostheses success.^6^

The determination of gaps in implant-supported rehabilitations have usually been described in the literature by two-dimensional (2D) techniques, where linear data were obtained by direct evaluation in a microscope or in embedded and sectioned specimens.^9^^,^^10^ 2D measurements of the gap in the implant-abutment connection has been reported to reach 60 μm.^10^ Although these techniques have been proven as reliable methods, most authors agree that these measurements generally involve human errors, which include non-standardized assessment sites, making the interpretation and comparison between studies a challenge.^11^^–^^13^ Nonetheless, methods measuring volume such as μCT have gained attraction as non-destructive techniques that allow for the three-dimensional (3D) qualitative and quantitative evaluation of implants and prosthetic components, providing a more thorough characterization of the abutment-implant interface.^9^ In the current study, the gap measured by 2D analyses of virtually-sectioned μCT reconstructed silicone replicas were consistent with those previously mentioned, which, along with the data of previous studies, may support its use as an alternative to reduce research time and costs.^5^

## Conclusion

The Control S.I.N and Singular Ti-base abutments showed improved volumetric and marginal fit relative to Odontofix and EFF. Therefore, some Ti-base abutments fabricated by companies other than the proprietary implant manufacturer may present more misfit.
